# The Minds That Matter: How Robots’ Mental Capacities Shape Children’s Evaluations and Trust

**DOI:** 10.1162/OPMI.a.37

**Published:** 2025-10-29

**Authors:** Anastasiia D. Grigoreva Crean, Arber Tasimi

**Affiliations:** Department of Psychology, Emory University, Atlanta, GA, USA

**Keywords:** mind perception, robots, epistemic trust, moral expertise, social evaluation, developmental psychology

## Abstract

Robots express a great deal of diverse human-like capacities, ranging from communicating in natural languages to displaying emotions to responding to physical touch. Here we examined the role of different kinds of mental capacities on children’s evaluations of, and trust in, robots. We presented 6- to 9-year-olds with identical-looking humanoid robots described as having one (or none) of the following capacities: cognitive-perceptual, social-emotional, or physiological. Across three studies (*N* = 287), we found that children differentially evaluated (Studies 1A and 1B) and selectively trusted (Study 2) robots with different types of minds. The diverging evaluations (i.e., of benevolence, intelligence, affinity, and epistemic appeal) of robots with different minds emerged between ages 7 and 8 and became stronger with age. Moreover, these differences translated into selective trust choices: children trusted robots with cognitive-perceptual capacities over robots with social-emotional capacities in a factual, but not a social, context, and over robots with bodily capacities across both contexts. Altogether, these findings open avenues for future interdisciplinary research on children’s reasoning about emerging technologies.

## INTRODUCTION

Robots, which are frequently endowed with artificial intelligence (AI), are becoming a part of everyday life, with children often being the main target of robotic innovation (Pearson, 2020). Perhaps unsurprisingly, the rise of child-friendly robots has spurred a wealth of research (for a review, see van Straten, Peter, & Kühne, 2020) on how children relate to (e.g., Severson & Carlson, 2010), interact with (e.g., Belpaeme et al., 2013), and learn from these cutting-edge technologies (e.g., Belpaeme, Kennedy, et al., 2018). Amidst such advancements, a key question arises: What kinds of intelligent machines are most likely to appeal to children? After all, child-friendly robots’ capacities range from responding to physical touch through expressive sounds and movements (e.g., Keepon by BeatBots) to recognizing objects and communicating via natural language (e.g., Nao by Softbank Robotics) to expressing empathy and emotional support (e.g., Moxie by Embodied). Here we investigate the role of different kinds of mental capacities on children’s social evaluations of, and trust in, robots.

Understanding children’s evaluations of robots as social and epistemic agents is a timely and pragmatically important issue. After all, most child-friendly robots are designed to address children’s social (Dawe et al., 2019; Pearson, 2020; Sharkey & Sharkey, 2010) and educational needs (for reviews, see Belpaeme, Kennedy, et al., 2018; Toh et al., 2016). Indeed, children already treat robots as informants (Breazeal et al., 2016) whose potential as educators ranges from relatively formal contexts, such as science (e.g., Hashimoto et al., 2013; Shiomi et al., 2015) or language learning (for reviews, see Belpaeme, Vogt, et al., 2018; Kanero et al., 2018) to creativity (Ali et al., 2019), curiosity (Gordon et al., 2015), and even empathy (Pashevich, 2022). Moreover, children tend to accept robots as friends (Beran, Ramirez-Serrano, Kuzyk, Fior, et al., 2011; Fior et al., 2010; Kahn et al., 2012; Manzi et al., 2020), treat them as social agents (Baxter et al., 2013; Desideri et al., 2021; Okanda et al., 2018), and even model altruistic and collaborative behaviors after them (Peter et al., 2021; Zaga et al., 2017).

A significant aspect of what enables robots to assume these various roles is their human-like capabilities. Correspondingly, children’s tendency to see robots as human-like has received considerable attention (e.g., Goldman et al., 2025; Kahn et al., 2011; Kim et al., 2019; Okanda et al., 2021; Tung, 2016). In general, children tend to attribute at least some human-like mental capacities to robots (e.g., Beran, Ramirez-Serrano, Kuzyk, Fior, et al., 2011; Kahn et al., 2012; Manzi et al., 2020; Okanda et al., 2021), which might have consequences for how children treat them (e.g., Flanagan et al., 2023; Reinecke et al., 2025; Sommer et al., 2019). However, much of this past work assessed children’s default assumptions of what kinds of mental capacities robots have (e.g., Flanagan et al., 2021, 2024; Kahn et al., 2012; Reinecke et al., 2025). Others measured children’s attributions of mental capacities to robots as a product of other features, for example, by manipulating how robots behave and/or look (e.g., Brink & Wellman, 2020; Caruana et al., 2023; Chernyak & Gary, 2016; Flanagan et al., 2023; Manzi et al., 2020). And of the studies that have examined robots’ perceived mind itself as a potential predictor of other attitudes, they treated it as a unitary phenomenon (Sommer et al., 2019; van Straten et al., 2023; van Straten, Peter, Kühne, et al., 2020), leaving open the question of whether the particular contents of robots’ minds influence children’s reasoning.

When it comes to perceiving the contents of others’ minds, which is known as *mind perception*, people do not simply view minds as one thing (i.e., as either present in an entity or not), but as a multi-dimensional phenomenon (Gray et al., 2007; Weisman et al., 2017b; Malle, 2019; but see Tzelios et al., 2022). According to one model of mind perception, the minds of others are perceived along three dimensions, which include cognitive-perceptual, social-emotional, and physiological capacities (Weisman et al., 2017a, 2017b); these capacities can be thought of as *Mind* (cognitive-perceptual), *Heart* (social-emotional), and *Body* (physiological). The *Mind* dimension includes capacities such as remembering things and making choices, the *Heart* dimension includes capacities such as feeling love and knowing right from wrong, and the *Body* dimension includes capacities such as feeling hungry or tired (Weisman et al., 2017b). Support for this model comes from developmental (Weisman et al., 2017a, 2018) and cross-cultural research (Weisman, Legare, et al., 2021).

Such work on mind perception offers an empirically grounded roadmap for investigating how children reason about and trust robots with various mental capacities. Here we capitalize on the period of development (ages 6 to 9) during which children begin to demonstrate an appreciation of different dimensions of mind (Weisman et al., 2017a, 2018) and domains of expertise (e.g., Danovitch & Keil, 2007, 2008) to examine whether the three dimensions of mind perception differentially inform children’s social and epistemic evaluations. In three studies, we presented 6- to 9-year-olds with identical-looking humanoid robots described as having different types of mind based on the three-dimensional mind perception model (Weisman et al., 2017a, 2017b). Our first goal was to examine whether different kinds of minds elicit different evaluations across a range of dimensions: similarity, affinity, benevolence, epistemic expertise (Studies 1A and 1B), and intelligence (Study 1B). Our second goal was to examine whether different kinds of minds influence children’s selective trust in two domains: factual knowledge and social imitation (Study 2).

The research procedures were approved by the Institutional Review Board at Emory University. We report how we determined our sample sizes, all data exclusions, manipulations, and measures in this paper. Study 1A and Study 2 were not preregistered and hence were largely exploratory; Study 1B was preregistered (https://aspredicted.org/kyqf-tgvr.pdf). Data were analyzed using R, version 4.3.1 (R Core Team, 2024). All data and code are available at: https://researchbox.org/4269.

## STUDY 1A

Study 1A tested whether there are systematic differences in children’s appraisals of robots with different types of mental capacities—*Mind*, *Heart*, and *Body* (Weisman et al., 2017a)—or lack thereof. We focused on children’s evaluations along four dimensions: *anthropomorphism* (i.e., a robot’s perceived similarity to themselves), *affinity* (i.e., desire to be friends with a robot), *benevolence* (i.e., a robot’s perceived niceness), and *epistemic expertise* (i.e., desire to learn from a robot).

### Methods

#### Participants.

The final sample consisted of 95 6–9-year-old children (*M*_*age*_ = 7.99, *SD*_*age*_ = 1.15, range = 6.04–9.92, 54 females, 40 males, 1 non-binary) recruited from Children Helping Science (Scott & Schulz, 2017). As reported by the participants’ caregivers, who were able to check multiple demographic categories, our sample was: 63% White, 31% Asian, 11% Hispanic, Spanish, or Latinx, 3% Black or African American, 3% Multiracial, 2% American Indian or Alaska Native, 1% Native Hawaiian or Pacific Islander, and 1% provided no answer. All children were in the United States and tested individually on Zoom. Parents of participating children gave written informed consent; children also provided oral assent.

Given the exploratory nature of this study and unknown effect sizes, we opted for a sample size that was determined by a stopping rule of 96 children. Two children were excluded and replaced because they did not pay attention. One additional child was excluded after data collection was completed because they fell outside of the selected age range, resulting in the final sample of 95 participants. A sensitivity analysis for a multiple linear regression using G*Power version 3.1.9.6 (Faul et al., 2007) indicated the smallest observable effect size with a sample of 95, power = .80, and alpha = .05 was *η*_p_^2^ = .11. To achieve a well-balanced sample, we aimed to recruit similar numbers of younger (47 6–7-year-olds) and older (48 8–9-year-olds) children.

#### Materials and Procedure.

**Figure 1.**
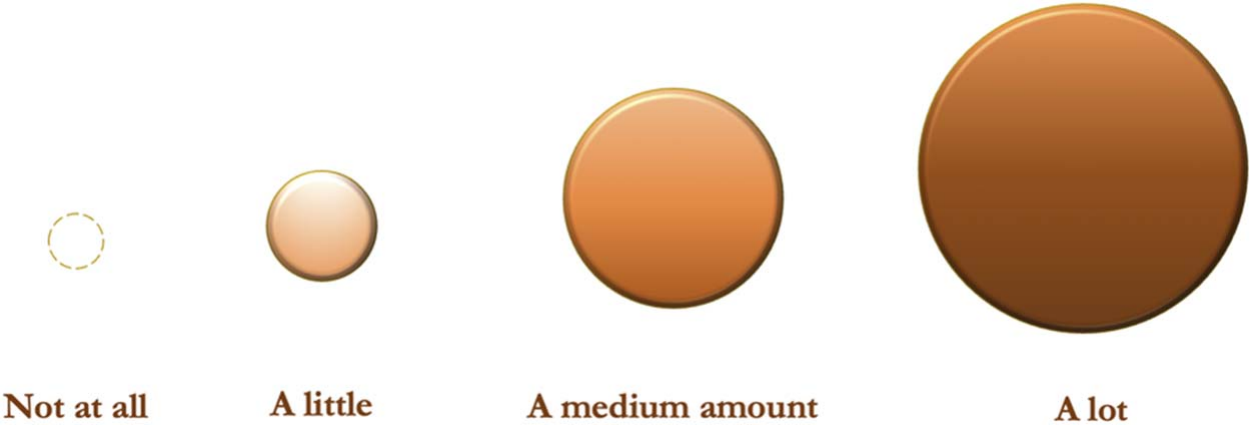
Child-Friendly Likert Scale in Studies 1A and 1B.

Participants were first introduced to a child-friendly Likert scale (Figure 1; adapted from Sommer et al., 2019), ranging from 0 (“not at all”) to 3 (“a lot”). They were familiarized with the scale through three questions that were unrelated to the study and chosen to elicit the range of scale options: “How much do you like candy?”, “How much do you like dog food?”, “How much do you like carrots?” (adapted from Tasimi & Gelman, 2021). These questions were asked at the beginning of the study.

**Figure 2.**
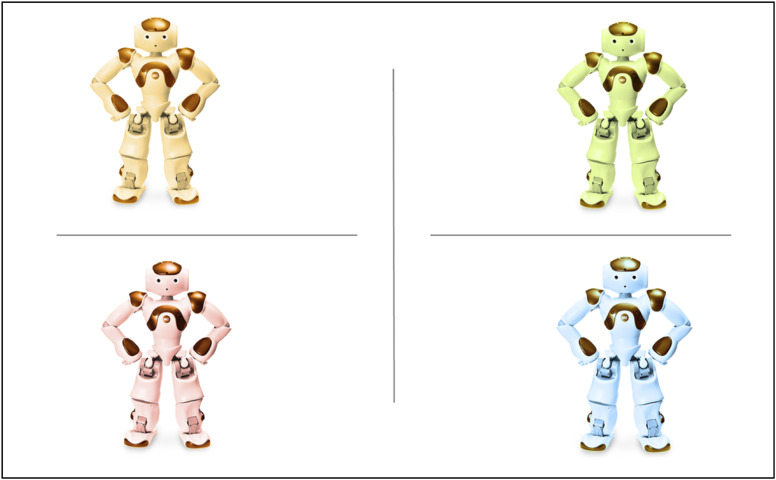
Pictures of the Robots Used in All Studies.

Participants were then shown pictures of four humanoid Nao robots which looked identical to each other, except for their color (Figure 2). Past work has shown that children are sensitive to how robots are framed to them (e.g., Beran, Ramirez-Serrano, Kuzyk, Nugent, et al., 2011; Chernyak & Gary, 2016; Ros et al., 2011; van Straten et al., 2023; van Straten, Peter, Kühne, et al., 2020; Vogt et al., 2017); thus, to directly assess the consequences of different kinds of mind, we explicitly described robots’ mental capacities to participants.

Each robot was described as having a different type of mind—*Mind*, *Heart*, or *Body*—except for the control robot (henceforth, *Control*), which was described as having no human-like mental capacities whatsoever (see Table 1 for robot descriptions). Participants were introduced to each robot, one at a time, and asked four questions about it using the Likert scale. The order and color of the robots as well as the order of the questions were counterbalanced across participants. The four questions were as follows: (1) How similar do you think this robot is to kids like you? (2) How much would you want to be friends with this robot? (3) How nice do you think this robot is? (4) How much would you like to learn from this robot?

**Table 1.** Robot Descriptions in Study 1A.

**Control**	**Mind**	**Heart**	**Body**
*No mental capacities*	*Cognitive-perceptual capacities*	*Social-emotional capacities*	*Physiological capacities*
This robot cannot do any of the things that you can do. It cannot feel things in its body. It cannot feel things in its heart. And it cannot understand things with its mind.	This robot can understand things with its **mind** like you. It can figure out how to do things and remember things. It can make choices. And it can talk to others. So, this robot can understand things with its mind like you, but in other ways, it’s not like you.	This robot can feel things in its **heart** like you. It can feel happy or sad. It can feel love. And it can know what’s nice and what’s mean. So, this robot can feel things in its heart like you, but in other ways, it’s not like you.	This robot can feel things in its **body** like you. It can get hungry. It can feel pain and feel tired. And it can feel sick. So, this robot can feel things in its body like you, but in other ways, it’s not like you.

### Results

To examine the effect of Mind Type (*Mind*, *Heart*, *Body*, or *Control*) on each rating (similarity, desire to be friends, niceness, desire to learn), we ran four separate linear mixed-effects models with Mind Type, participant age (in years), and their interaction as fixed effects, and random intercepts for participant to account for individual variability in baseline ratings. Wald chi-square analysis of variance was applied to each model to assess the significance of its predictors. Significant main effects were followed up with pairwise estimated marginal means comparisons with a Benjamini-Hochberg correction. To probe significant interactions, this correction was also applied to simple slopes analyses and estimated marginal means comparisons at each 6-month increment in participant age. This approach was a compromise between detail and manageability: it allowed us to examine a more fine-grained developmental progression of relying on full years while maintaining a limited number of pairwise comparisons for ease of interpretation at specific developmental points.

#### Similarity.

**Figure 3.**
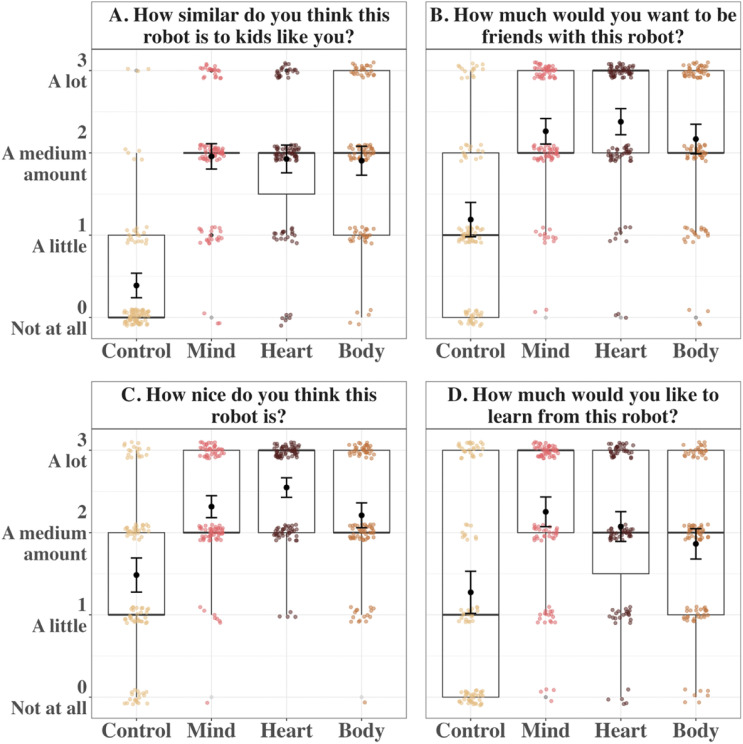
Mean Ratings of All Variables in Study 1A. *Note*. Boxplots and individual points show the distribution of children’s responses to each of four questions for the four mind types in Study 1A. Error bars are 95% CIs around the mean.

There was a significant main effect of Mind Type on robots’ perceived similarity, *χ*^2^(3) = 308.91, *p* < .001 (Figure 3A). There was no significant main effect of age, *χ*^2^(1) = 1.07, *p* = .301, nor was there a significant interaction between Mind Type and age, *χ*^2^(3) = 4.12, *p* = .249.

Any kind of mind increased children’s ratings of robots’ perceived similarity to themselves (*Mind*: *M* = 1.96, *SD* = 0.76, *d* = 1.47; *Heart*: *M* = 1.93, *SD* = 0.83, *d* = 1.36; *Body*: *M* = 1.91, *SD* = 0.86, *d* = 1.35) compared to the *Control* robot (*M* = 0.39, *SD* = 0.73), *p*s_adj_ < .001. There were no differences among the robots with “minds,” *p*s_adj_ > 0.84.

#### Friendship.

There was a significant main effect of Mind Type on children’s desire to be friends with a robots, *χ*^2^(3) = 165.78, *p* < .001 (Figure 3B). Once again, there was no significant main effect of age, *χ*^2^(1) = 0.34, *p* = .558, nor was there a significant interaction between Mind Type and age, *χ*^2^(3) = 4.03, *p* = .258.

Any kind of mind increased children’s desire to be friends with robots (*Mind*: *M* = 2.26, *SD* = 0.76, *d* = 1.01; *Heart*: *M* = 2.38, *SD* = 0.77, *d* = 1.04; *Body*: *M* = 2.17, *SD* = 0.88, *d* = 0.93) compared to the *Control* robot (*M* = 1.19, *SD* = 1.02), *p*s_adj_ < .001; there were no differences among the robots with “minds,” *p*s_adj_ > .06.

#### Niceness.

There again was a significant main effect of Mind Type on ratings of robots’ niceness, *χ*^2^(3) = 110.09, *p* < .001 (Figure 3C). There was no significant main effect of age, *χ*^2^(1) = 0.68, *p* = .408, nor was there a significant interaction between Mind Type and age, *χ*^2^(3) = 0.17, *p* = .983.

Any kind of mind increased children’s ratings of robots’ niceness (*Mind*: *M* = 2.32, *SD* = 0.66, *d* = 0.77; *Heart*: *M* = 2.55, *SD* = 0.58, *d* = 0.87; *Body*: *M* = 2.21, *SD* = 0.74, *d* = 0.62) compared to the *Control* robot (*M* = 1.48, *SD* = 1.02), *p*s_adj_ < .001. Out of the robots with “minds,” children rated the *Heart* robot as nicer than both the *Mind* robot, *p*_adj_ = .038, *d* = 0.26, and the *Body* robot, *p*_adj_ = .003, *d* = 0.37, while there was no difference between the *Mind* and *Body* robots, *p*_adj_ = .327.

#### Learning.

Although there was no significant main effect of age *χ*^2^(1) = 2.29, *p* = .131, there was a significant main effect of Mind Type on children’s desire to learn from robots, *χ*^2^(3) = 70.38, *p* < .001 (Figure 3D). Any kind of mind increased children’s desire to learn from robots (*Mind*: *M* = 2.25, *SD* = 0.89, *d* = 0.71; *Heart*: *M* = 2.07, *SD* = 0.89, *d* = 0.60; *Body*: *M* = 1.86, *SD* = 0.91, *d* = 0.45) compared to the *Control* robot (*M* = 1.27, *SD* = 1.26), *p*s_adj_ < .001. Moreover, children were more willing to learn from the *Mind* robot than the *Body* robot, *p*_adj_ = .003, *d* = 0.37. However, there was no difference between the *Mind* and *Heart* robots, *p*_adj_ = .151, or between the *Heart* and *Body* robots, *p*_adj_ = .110.

**Figure 4.**
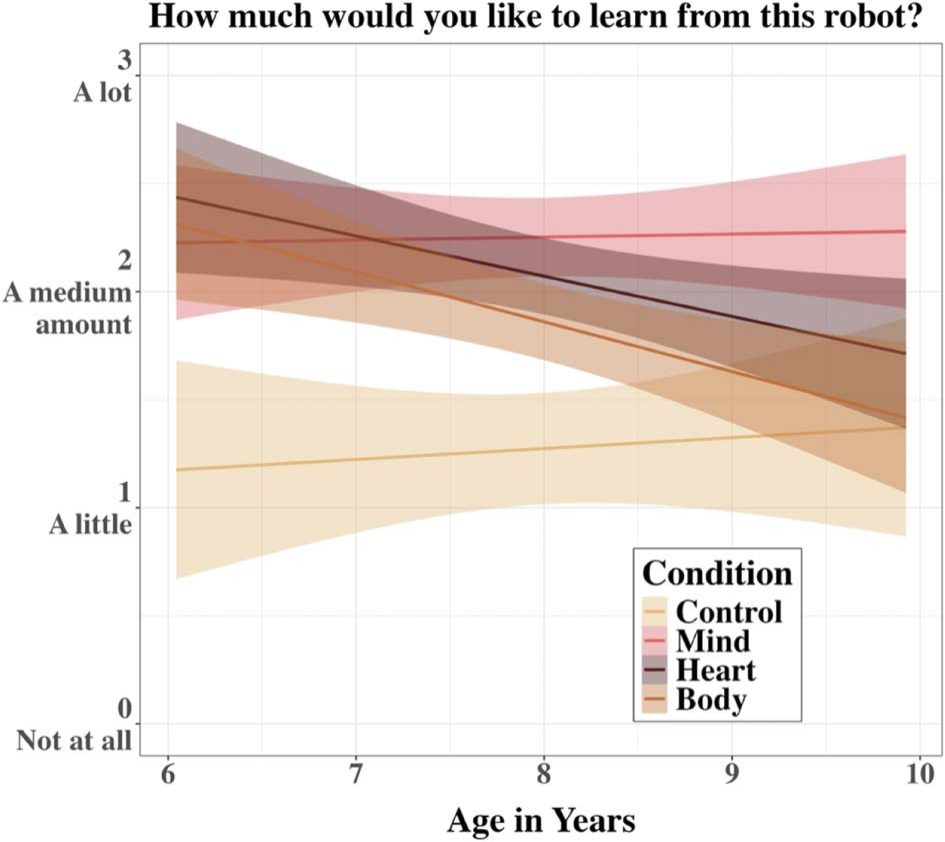
Learning Ratings by Mind Type and Participant Age in Study 1A. *Note*. A linear graph shows mind type effects by participant age for children’s desire to learn from a robot in Study 1A. Errors around the regression lines are 95% CIs.

The main effect of Mind Type was qualified by a significant interaction between Mind Type and participant age, *χ*^2^(3) = 10.14, *p* = .017 (Figure 4). An estimated marginal means analysis across the levels of Mind Type at each 6-month increment in age showed that a preference to learn from the *Mind* robot over the *Body* robot emerged with age. Children younger than age 8 did not show a significant difference between the *Mind* and *Body* robots, *p*s_adj_ > .18, whereas children aged 8 and older indicated a greater desire to learn from the *Mind* robot compared to the *Body* robot, *p*s_adj_ < .010. Moreover, starting at age 9, the *Body* robot was not different from the *Control* robot, *p*s_adj_ > .25, and at age 9.5, the *Heart* robot was also no longer different from the *Control* robot, *p*s_adj_ = .14. A simple slope analysis revealed a significant negative linear relationship between age and desire to learn from the *Body* robot, *β* = −0.23, *SE* = 0.09, *p*_adj_ = .038. There was no significant effect of age for the *Control*, *Mind*, and *Heart* robots, *p*s_adj_ > .071. Thus, the growing difference between the *Mind* and *Body* robots is best explained by the declining desire to learn from the *Body* robot relative to a stable one for the *Mind* robot.

### Discussion

Having any kind of human-like mind increased children’s perceptions of robots’ similarity to themselves and affinity for robots. By contrast, some types of mind mattered more than others when it came to children’s explicit evaluations of robots’ benevolence (i.e., the *Heart* robot was rated as nicer than all other robots) and epistemic expertise (i.e., children indicated greater desire to learn from the *Mind* robot than the *Body* robot but not the *Heart* robot).

That said, these findings are limited by several methodological choices. First, except for the *Control* robot, the descriptions for the three key robots (*Mind*, *Heart*, *Body*) did not specify what the robots could *not* do; instead, they only highlighted what the robots were capable of doing. It is possible, then, that children assumed that the robots had additional capacities beyond those mentioned in the description. Indeed, this might explain some of the null effects, such as a lack of a difference between the *Heart* and *Body* robots when it came to children’s desire to learn from them. Second, there were no comprehension checks to determine whether children understood that each robot possessed or lacked particular mental capacities. Finally, although children’s desire to learn from a robot may reflect their assessments of a robot’s intelligence, it might also be sensitive to other factors, such as perceived benevolence, which has been documented in prior work (e.g., Johnston et al., 2015; Landrum et al., 2013; Lane et al., 2013). A more explicit evaluation of robots’ intelligence could be informative to draw parallels with children’s assessments of robots’ benevolence, as both traits have been shown to influence children’s tendency to trust others (Johnston et al., 2015; Landrum et al., 2013; Lane et al., 2013). All these shortcomings were addressed in a preregistered replication, which we report below.

## STUDY 1B

We preregistered a replication of Study 1A (https://aspredicted.org/kyqf-tgvr.pdf) that used less ambiguous stimuli and implemented comprehension checks. The goal of Study 1B was to replicate systematic differences in children’s evaluations of robots with different types of mental capacities—*Mind*, *Heart*, and *Body—*or lack thereof. In addition to the four evaluations used in Study 1A (similarity, affinity, benevolence, and epistemic expertise), Study 1b also asked children to evaluate robots’ intelligence (i.e., how smart they thought each robot was).

### Methods

#### Participants.

The final sample consisted of 96 6–9-year-old children (*M*_*age*_ = 8.06, *SD*_*age*_ = 1.27, range = 6.00–9.96, 62 females, 34 males) recruited from Children Helping Science (*n* = 25), Emory University’s Child Study Center (*n* = 25), and at an elementary school in suburban Connecticut (*n* = 46). We followed the same testing and consent procedures as in Study 1a. Target sample size of 96 children was selected and pre-reregistered to match Study 1a. Two children were excluded and replaced because they failed a single comprehension check trial more than twice (a preregistered exclusion criterion). Once again, we aimed for a well-balanced sample of younger (46 6–7-year-olds) and older children (50 8–9-year-olds). As reported by the participants’ caregivers, who were able to check multiple demographic categories, our sample was: 78% White, 25% Asian, 5% Hispanic, Spanish, or Latinx, 5% Black or African American, 5% Multiracial, and 3% said “Other” or provided no answer.

#### Materials and Procedure.

The materials and procedure were identical to Study 1a with three exceptions. First, the *Mind*, *Heart*, and *Body* robot descriptions included not only what the robots could do but also what they could not do (see Table 2). Second, each robot description was followed by three comprehension check questions presented in a counterbalanced order (“Can this robot feel things in its body? Can this robot feel things in its heart? Can this robot understand things with its mind?”), which required participants to answer “yes” or “no.” Incorrect answers were corrected (see Table S1 in the Supplementary Materials for an overview of children’s performance on these comprehension questions), and the question was repeated up to two times (e.g., “Actually, this robot cannot feel things in its body. Let me ask you again: can this robot feel things in its body?”). If a participant answered incorrectly more than twice to a single comprehension question, the study ended early, and the participant was excluded from the study (*n* = 2). Third, we added a fifth question about each robot’s intelligence: How smart do you think this robot is? All five questions were counterbalanced across participants.

**Table 2.** Robot Descriptions in Study 1B.

**Control**	**Mind**	**Heart**	**Body**
*No mental capacities*	*Cognitive-perceptual capacities*	*Social-emotional capacities*	*Physiological capacities*
This robot cannot do any of the things that you can do. It cannot feel things in its body. It cannot feel things in its heart. And it cannot understand things with its mind.	This robot can understand things with its **mind** like you. It can figure out how to do things and remember things. It can make choices. And it can talk to others. But in other ways, this robot is not like you. It cannot feel things in its heart. And it cannot feel things in its body. So, this robot can understand things with its mind, but it can’t feel things in its heart or feel things in its body.	This robot can feel things in its **heart** like you. It can feel happy or sad. It can feel love. And it can know what’s nice and what’s mean. But in other ways, this robot is not like you. It cannot feel things in its body. And it cannot understand things with its mind. So, this robot can feel things in its heart like you, but it can’t feel things in its body or understand things with its mind.	This robot can feel things in its **body** like you. It can get hungry. It can feel pain and feel tired. And it can feel sick. But in other ways, this robot is not like you. It cannot feel things in its heart. And it cannot understand things with its mind. So, this robot can feel things in its body like you, but it can’t feel things in its heart or understand things with its mind.

### Results

The analytical strategy was identical to Study 1A.

#### Similarity.

**Figure 5.**
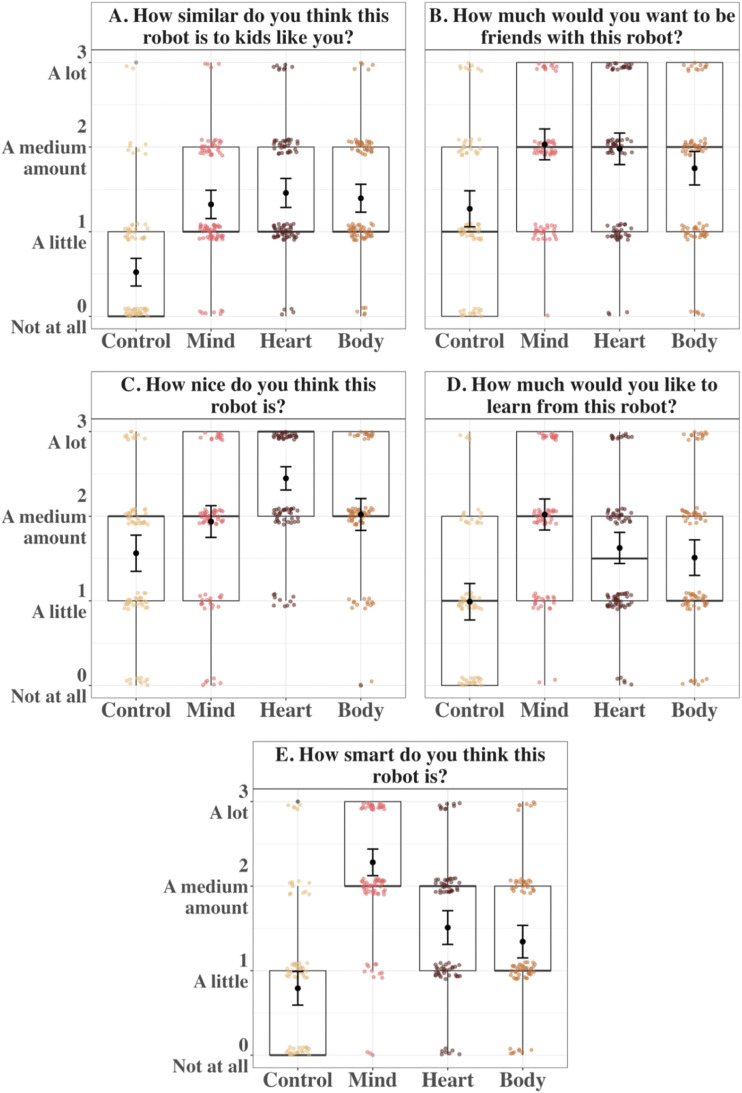
Mean Ratings of All Variables in Study 1B. *Note*. Boxplots and individual points show the distribution of children’s responses to each of five questions for the four mind types in Study 1B. Error bars are 95% CIs around the mean.

Although there was no significant main effect of age, *χ*^2^(1) = 3.14, *p* = .077, there was a significant main effect of Mind Type on robots’ perceived similarity, *χ*^2^(3) = 121.90, *p* < .001 (Figure 5A). Any kind of mind increased children’s ratings of robots’ perceived similarity (*Mind*: *M* = 1.32, *SD* = 0.83, *d* = 0.83; *Heart*: *M* = 1.46, *SD* = 0.85, *d* = 0.95; *Body*: *M* = 1.40, *SD* = 0.81, *d* = 0.90) compared to the *Control* robot (*M* = 0.52, *SD* = 0.81), *p*s_adj_ < .001. There were no differences among the robots with different “minds,” *p*s_adj_ > 0.24.

**Figure 6.**
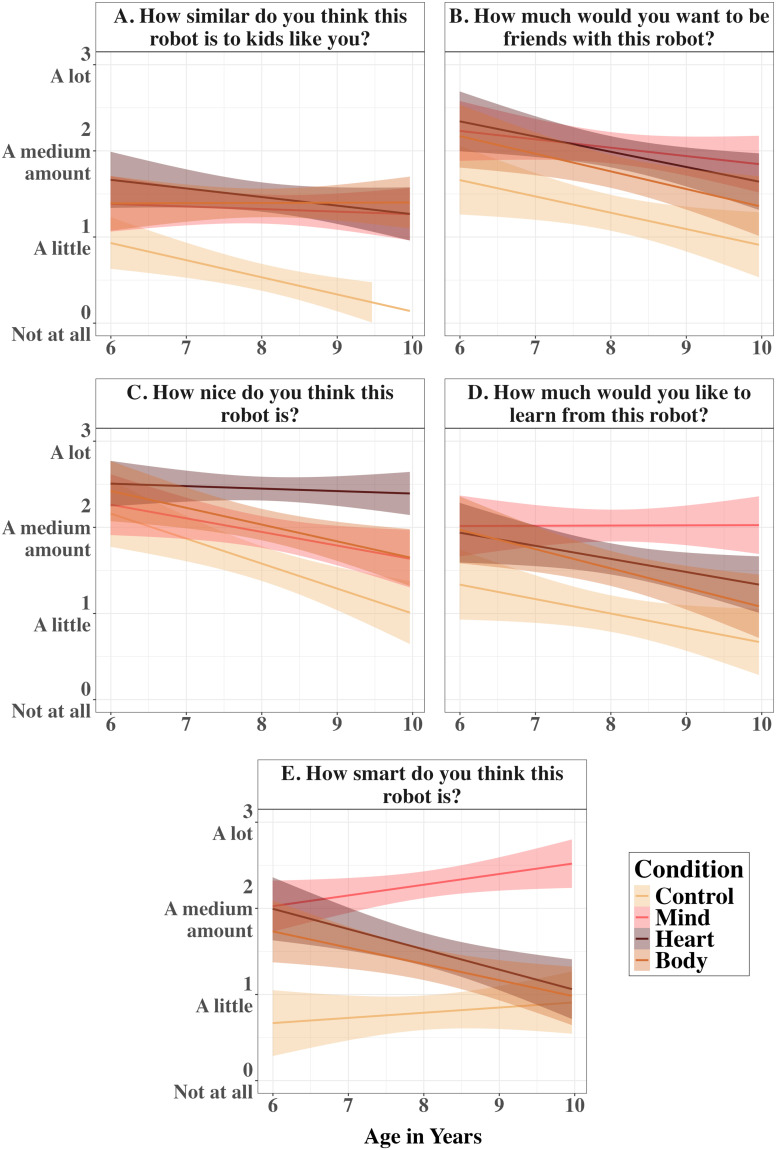
Ratings by Mind Type and Participant Age for all Variables in Study 1B. *Note*. Linear graphs show mind type effects by participant age for all five variables in Study 1B. Errors around the regression lines are 95% CIs.

This main effect of Mind Type was qualified by a significant interaction between Mind Type and age, *χ*^2^(3) = 7.96, *p* = .047 (Figure 6A). To probe this interaction, we ran simple slopes analyses for age within each Mind Type. There was a significant negative linear relationship between age and similarity ratings for the *Control* robot, *β* = −0.20, *SE* = 0.07, *p* = .011. Age was not related to the ratings for any of the “mindful” robots, *p*s_adj_ > 0.26.

#### Friendship.

There was no significant interaction between Mind Type and age, *χ*^2^(3) = 1.98, *p* = .577. However, there was a significant main effect of age on children’s desire to be friends with robots, *χ*^2^(3) = 8.69, *p* = .003, such that older children were less willing to befriend robots than younger children, *β* = −0.19, *SE* = 0.08, *p* = .014 (Figure 6B).

There was also a significant main effect of Mind Type, *χ*^2^(1) = 65.13, *p* < .001 (Figure 5B). Any kind of mind increased children’s desire to be friends with robots (*Mind*: *M* = 2.03, *SD* = 0.90, *d* = 0.70; *Heart*: *M* = 1.98, *SD* = 0.92, *d* = 0.64; *Body*: *M* = 1.75, *SD* = 0.97, *d* = 0.47) compared to the *Control* robot (*M* = 1.27, *SD* = 1.05), *p*s_adj_ < .001. Moreover, children were more willing to be friends with the *Mind* robot than the *Body* robot, *p*_adj_ = .012, *d* = 0.32, and the *Heart* robot than the *Body* robot, *p*_adj_ = .036, *d* = 0.22. There was no difference between the *Mind* and *Heart* robots, *p*_adj_ = .621.

#### Niceness.

Again, there was a significant main effect of age on children’s ratings of robots niceness, *χ*^2^(1) = 11.65, *p* < .001, such that niceness ratings decreased with age, *β* = −0.29, *SE* = 0.07, *p* < .001 (Figure 6C). There was also a significant main effect of Mind Type, *χ*^2^(3) = 70.61, *p* < .001 (Figure 5C). Any kind of mind increased children’s ratings of robots niceness (*Mind*: *M* = 1.94, *SD* = 0.93, *d* = 0.36; *Heart*: *M* = 2.45, *SD* = 0.68, *d* = 0.81; *Body*: *M* = 2.02, *SD* = 0.93, *d* = 0.43) compared to the *Control* robot (*M* = 1.56, *SD* = 1.05), *p*s_adj_ < .001. Out of the robots with “minds,” children rated the *Heart* robot as nicer than both the *Mind* robot, *p*_adj_ < .001, *d* = 0.45, and the *Body* robot, *p*_adj_ < .001, *d* = 0.43. There was no difference between the *Mind* and *Body* robots, *p*_adj_ = .432.

These main effects were qualified by a significant interaction between Mind Type and age, *χ*^2^(3) = 9.92, *p* = .019 (Figure 6C). A comparison of estimated marginal means across the levels of Mind Type at each 6-month increment in age revealed a developmental trend: starting at 6.5 years old, children rated the *Heart* robot as significantly nicer than the *Control* robot, *p*s_adj_ < .029; at age 7, they started to rate the *Heart* robot as nicer than the Mind robot, *p*s_adj_ < .021; and finally, starting at age 7.5, they rated the *Heart* robot as nicer than the *Body* robot, *p*s_adj_ < .006.

A simple slopes analysis revealed a significant negative relationship between age and niceness for the *Control* robot, *β* = −0.29, *SE* = 0.07, *p*_adj_ < .001, the *Mind* robot, *β* = −0.16, *SE* = 0.07, *p*_adj_ = .036, and the *Body* robot *β* = −0.19, *SE* = 0.07, *p*_adj_ = .013. In contrast, age was not related to niceness for the *Heart* robot, *p*_adj_ = .687. Thus, the growing difference between the *Heart* robot and other robots is best explained by children’s declining perceptions of the *Control*, *Mind*, and *Body* robots’ niceness relative to the stable perceptions of the *Heart* robot’s niceness.

#### Learning.

There was no significant interaction between Mind Type and age, *χ*^2^(3) = 7.62, *p* = .055, but there was a significant main effect of age on children’s desire to learn from robots, *χ*^2^(1) = 5.48, *p* = .019, such that children’s desire to learn from robots decreased with age, *β* = −0.17, *SE* = 0.08, *p* = .033 (Figure 6D).

There was also a significant main effect of Mind Type, *χ*^2^(3) = 92.52, *p* < .001 (Figure 5D). Any kind of mind increased children’s desire to learn from robots (*Mind*: *M* = 2.02, *SD* = 0.91, *d* = 0.86; *Heart*: *M* = 1.62, *SD* = 0.91, *d* = 0.70; *Body*: *M* = 1.51, *SD* = 1.04, *d* = 0.53) compared to the *Control* robot (*M* = 0.99, *SD* = 1.06), *p*s_adj_ < .001. Moreover, children were more willing to learn from the *Mind* robot compared to the *Body* robot, *p*_adj_ < .001, *d* = 0.42, and compared to the *Heart* robot, *p*_adj_ < .001, *d* = 0.33. As in Study 1a, there was no difference between the *Heart* and *Body* robots, *p*_adj_ = .291.

#### Smartness.

There was a significant main effect of Mind Type on children’s ratings of robots’ smartness, *χ*^2^(3) = 182.76, *p* < .001 (Figure 5E). Any kind of mind increased children’s ratings of robots’ smartness (*Mind*: *M* = 2.28, *SD* = 0.78, *d* = 1.25; *Heart*: *M* = 1.51, *SD* = 0.98, *d* = 0.66; *Body*: *M* = 1.34, *SD* = 0.95, *d* = 0.50) compared to the *Control* robot (*M* = 0.79, *SD* = 0.98), *p*s_adj_ < .001. Out of the robots with “minds,” children rated the *Mind* robot as smarter than both the *Heart* robot, *p*_adj_ < .001, *d* = 0.61, and the *Body* robot, *p*_adj_ < .001, *d* = 0.78. There was no difference between the *Heart* and *Body* robots, *p*_adj_ = .136.

Although there was no significant main effect of age, *χ*^2^(1) = 1.45, *p* = .229, the main effect of Mind Type was qualified by a significant interaction between Mind Type and age, *χ*^2^(3) = 24.48, *p* < .001 (Figure 6E). A comparison of estimated marginal means across the levels of Mind Type at each 6-month increment in age revealed a developmental trend: starting at age 6, children rated the *Mind* robot as significantly smarter than the *Control* robot, *p*s_adj_ < .001; at age 6.5, they started to rate the *Mind* robot as smarter than the *Body* robot, *p*s_adj_ < .018; and finally, at age 7, they began to rate the *Mind* robot as smarter than the *Heart* robot, *p*s_adj_ = .010. Moreover, at age 9.5, children no longer viewed the *Heart* robot, *p*_adj_ = .128, or the *Body* robot, *p*_adj_ = .308, as smarter than the *Control* robot.

Analyses of simple slopes revealed a statistically significant negative linear relationship between age and smartness for the *Heart* robot, *β* = −0.24, *SE* = 0.07, *p*_adj_ = .006, and for the *Body* robot, *β* = −0.19, *SE* = 0.07, *p*_adj_ = .022. In contrast, the linear relationship between age and smartness was not statistically significant for the *Control* or *Mind* robots, *p*s_adj_ > .12. Thus, the growing difference between the *Mind* robot and other robots is best explained by children’s declining perceptions of the *Body* and *Heart* robots’ smartness relative to the stable perceptions of the *Mind* robot’s smartness.

### Discussion

Not only did Study 1B replicate the key findings of Study 1A, but it also revealed new differences along with several developmental trends. As in Study 1A, children rated all “mindful” robots as more similar to themselves than the *Control* robot. Extending the findings of Study 1A, Study 1B showed that children were more willing to become friends with the *Mind* and *Heart* robots than the Body robot. This new difference, which was not present in Study 1A, is likely due to the improved design of Study 1B, as it clarified each robot’s limitations and ensured children’s comprehension of what the robots could and could not do. Consistent with the results of Study 1A, the *Heart* robot was once again rated as the nicest while the *Mind* robot was rated as the most desirable to learn from. As for intelligence, which was included here but not in Study 1A, children rated the *Mind* robot as the smartest of all robots.

Overall, and taken together with Study 1A, there was a consistent developmental trajectory to three diverging evaluations (i.e., benevolence, intelligence, and epistemic appeal): children started to appreciate the distinctions between different kinds of minds between 7 and 8 years of age. Such evaluations can have far-reaching consequences, especially with respect to two areas for which child-friendly robots are designed: educational and social purposes (e.g., Belpaeme, Kennedy, et al., 2018; Dawe et al., 2019). Among the questions that these findings invite is: What kinds of robots would children trust in different contexts?

## STUDY 2

In Studies 1A and 1B, different types of mind influenced children’s views of robots as benevolent and intelligent as well as children’s desire to befriend and learn from them. Building on these findings, Study 2 tested whether such explicit evaluations translated into children’s epistemic and social trust in robots by asking children to engage in a selective word learning or a hypothetical social imitation task. On the one hand, children might show a global preference for one kind of robot. Indeed, past work has shown that children’s positive assumptions about knowledge and character cluster together (e.g., Brosseau-Liard & Birch, 2010; Danovitch & Keil, 2007; Lane et al., 2013; Stipek & Daniels, 1990), testifying to a “halo effect.” If this is the case, then children might trust either the *Mind* or the *Heart* robot across the board given their explicit evaluations of the *Mind* robot as intelligent and desirable to learn from and of the *Heart* robot as benevolent.

On the other hand, children may trust different robots on different issues. Children start to understand that separate domains of knowledge call for different experts during the period of development examined in our studies (e.g., Danovitch & Keil, 2004, 2007, 2008; Keil et al., 2008; Landrum & Mills, 2015). Both benevolence and social-emotional competence may be central to perceived expertise in the social domain (Danovitch & Keil, 2007, 2008), leading to the *Heart* robot being seen as a social expert whose actions are worth imitating more so than those of the other robots. Simultaneously, the *Mind* robot may be selectively trusted about novel labels due to its perceived competence and intelligence, which are important for children’s learning in more factual domains (Danovitch & Keil, 2007, 2008; Johnston et al., 2015).

Here we sought to explore these diverging possibilities and examine how children’s selective trust develops. Indeed, the results of Study 1A and Study 1B suggested that the differences in children’s explicit evaluations of the “mindful” robots emerge between 7 and 8 years of age, which is consistent with previous work showing that 7‒ to 9-year-olds are able to differentiate three dimensions of mind perception (Weisman et al., 2017a, 2018). Since children of these ages are also able to differentiate domains of expertise (Danovitch & Keil, 2004, 2007, 2008; Keil et al., 2008; Landrum & Mills, 2015), Study 2 examined the development of children’s selective trust in different robots, by pitting the *Mind*, *Heart*, and *Body* robots against each other in factual (i.e., object labels) and social (i.e., sharing behaviors) contexts. In both tasks, we aimed to move beyond simple identification of experts (e.g., Danovitch & Keil, 2007, 2008) to understand whether robots’ mental capacities have more meaningful consequences for children’s testimony endorsement and hypothetical social acts.

### Methods

#### Participants.

The final sample consisted of 96 6–9-year-old children (*M*_*age*_ = 7.86, *SD*_*age*_ = 1.22, range = 6.00–9.95, 47 females, 49 males) recruited from Children Helping Science (Scott & Schulz, 2017). We followed the same testing and consent procedure as in Studies 1A and 1B. As reported by the participants’ caregivers, who were able to check multiple demographic categories, our sample was: 69% White, 32% Asian, 9% Hispanic, Spanish, or Latinx, 2% Multiracial, 1% Native Hawaiian or Pacific Islander, 1% “other,” and 1% provided no answer. Since G*Power does not allow to compute an *a priori* power analysis for a logistic multiple regression, sample size was determined by an *a priori* power analysis for an exact proportion test (difference from constant) in G*Power using a medium effect size *g* = 0.15 and alpha = .05, which suggested the need for at least 90 participants to achieve an 80% power. To achieve a well-balanced sample, we recruited 48 6–7-year-olds and 48 8–9-year-olds.

#### Materials and Procedure.

Study 2 relied on the paradigm from Study 1A. Thus, the robot descriptions that were used can be found in Table 1. Each participant was assigned to either a Factual (*n* = 48) or a Social (*n* = 48) condition. In each condition, participants were shown pictures of the three humanoid robots from Studies 1A and 1B: *Mind*, *Heart*, or *Body*. Studies 1A and 1B indicated that robots with any kind of mind were evaluated as better epistemic and social agents than *Control*. Therefore, *Control* was excluded here to focus on the differences between robots with different mental capacities. As in Studies 1A and 1B, the experimenter emphasized the words “mind,” “heart,” and “body” when describing the robots to draw participants’ attention to the different types of mental capacities. Correspondingly, when participants were asked to choose between the robots during test tasks, the experimenter referred to the robots as “the Mind robot,” “the Heart robot,” and “the Body robot” (e.g., “Would you give the same number of cookies that the Body robot gave or would you give the same number of cookies that the Heart robot gave?”).

In the Factual condition, participants were told that the robots wanted to tell them about different things that were new. We used a selective trust paradigm inspired by previous work (e.g., Brink & Wellman, 2020; Koenig et al., 2004). This paradigm requires a participant to choose between conflicting testimonies of two informants. Various characteristics of informants (e.g., accuracy, similarity, benevolence) can be manipulated to assess their contributions to children’s epistemic trust (Marble & Boseovski, 2020; Mills, 2013). Here, we manipulated the kind of mind that each robotic informant possessed (*Mind*, *Heart*, or *Body*). For each pair of robots, participants were shown novel objects labeled differently by each robot (e.g., “The *Heart* robot says that this thing is called a modi. The *Mind* robot says that this thing is called a toma.”). The novel objects and names were selected from the NOUN database (Horst & Hout, 2016), similar to previous work (Brink & Wellman, 2020). After each testimony, participants were asked what they thought the object was called (e.g., “What do you think this thing is called: a modi like the *Heart* robot said or a toma like the *Mind* robot said?”). If a participant failed to choose, the experimenter repeated the question until a choice was made. There were three novel objects per each pair of robots; thus, each participant provided a total of nine selective trust judgments.

In the Social condition, participants were first told about a community box: “In this game, you will hear about different kinds of robots giving different amounts of things to a community box. The community box is used for kids who don’t have anything—no toys, no stickers, and no cookies. These kids like toys, stickers, and cookies just any other kids, but they just don’t have any of them.” Then, participants were presented with the same pairs of robots like in the Factual condition and were told that the robots varied in their levels of generosity toward a community box (e.g., “The *Heart* robot gave one amount of stickers and the *Mind* robot gave another amount of stickers.”). To avoid biasing children toward particular amounts, we did not specify the number of items that the robots initially had, nor the number of items they gave away. Then participants were asked to imagine that they had the same number of items as the robots and choose which robot’s sharing behavior they wanted to imitate (e.g., “Would you give the same number of stickers that the *Heart* robot gave, or would you give the same number of stickers that the *Mind* robot gave?”). There were three types of items (stickers, toys, cookies) per pair; thus, each participant provided a total of nine selective social trust judgments.

The order of pairs, colors of the robots, and the sides of each Mind Type were counterbalanced across the participants in both conditions.

### Results

Each participant made three selective trust judgments for each pair of robots (*Heart* vs. *Body*, *Mind* vs. *Body*, *Mind* vs. *Heart*) coded as 0 if they chose one robot or 1 if they chose another. The specific coding was distinct for each pair (e.g., *Body* = 0 and *Mind* = 1 in the *Body* vs. *Mind* pair; *Heart* = 0 and *Mind* = 1 in the *Heart* vs. *Mind* pair), resulting in a separate dependent variable for each pair. This precluded omnibus analyses or direct statistical comparisons across pairs. Therefore, we ran a separate logistic mixed-effects model for each pair. Each model included Condition (Factual vs. Social), participant age (in years), and their interaction as fixed effects. The random effects structure for each model included a random intercept for participant and a random intercept for trial nested within Condition. To account for multiple comparisons across the three models, a Benjamini-Hochberg correction was applied to the nine *p*-values derived from the fixed effects (main effects of Condition and Age, and their interaction) across the three separate analyses.

#### Mind vs. Heart.

Although there was no significant effect of age, *χ*^2^(1) = 1.08, *p*_adj_ = .533, there was a significant effect of Condition, *χ*^2^(1) = 12.32, *p*_adj_ = .004, indicating that participants were more likely to choose the *Mind* robot over the *Heart* robot in the Factual than in the Social condition, OR = 2.49, 95% CI [1.53, 4.04], *z* = 3.69, *p* < .001.

**Figure 7.**
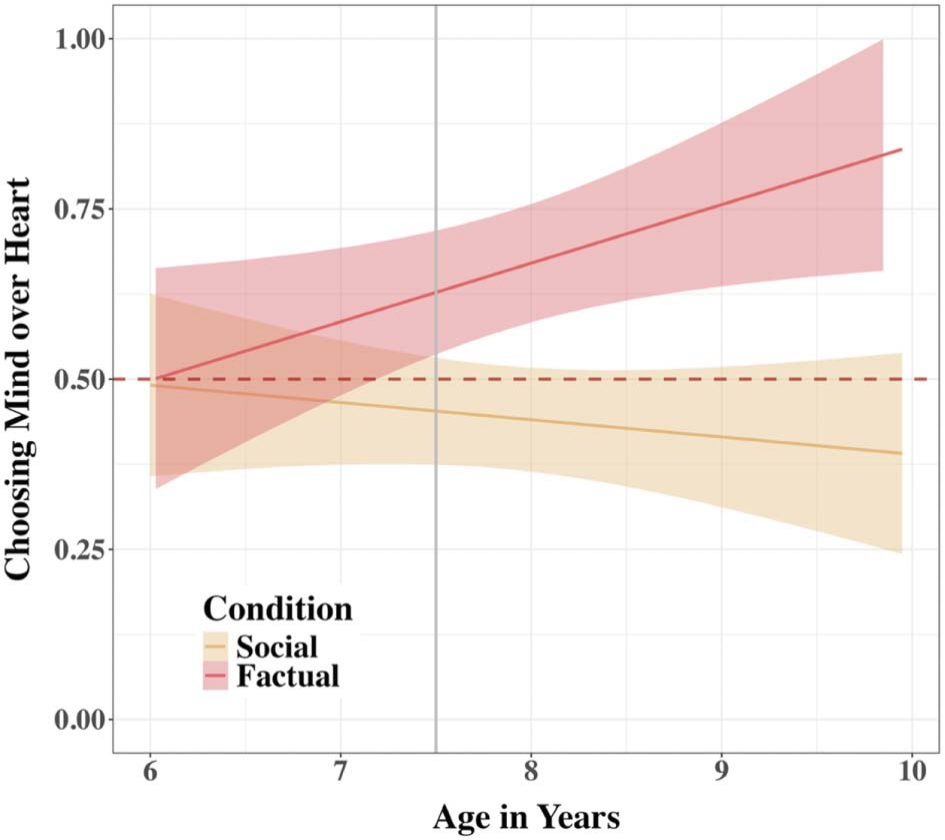
Tendency to Choose Mind over Heart by Condition and Participant Age in Study 2. *Note*. A linear graph shows an interaction between Condition and age in children’s tendency to trust the *Mind* robot over the *Heart* robot, averaged across trials in Study 2. Errors around the regression lines are 95% CIs. The horizontal line at 0.5 indicates a chance level of trust. The vertical line at 7.5 indicates the start of the significant difference between Conditions.

This main effect was qualified by a significant Condition x age interaction, *χ*^2^(1) = 5.75, *p*_adj_ = .0495, indicating that the difference between the conditions became more pronounced with age, OR = 1.64, 95% CI [1.09, 2.45] (Figure 7). To elucidate the significant Condition × age interaction, we examined the linear trend of participant age on the log-odds of choosing the *Mind* robot over the *Heart* robot separately for each condition using estimated marginal slopes. In the Factual condition, there was a significant positive effect of age; for each additional year of age, the log-odds of choosing the *Mind* robot increased by 0.39, *SE* = 0.16, *z* = 2.50, *p*_adj_ = .025 (OR = 1.48 95% CI [1.09, 2.00]). Conversely, in the Social condition, age did not have a statistically significant effect on the likelihood of choosing the *Mind* robot, *p*_adj_ = .441. The difference between these two age trends was statistically significant, *p* = .017, confirming that the developmental trajectory of choice preference for the *Mind* robot over the *Heart* robot differed significantly between the Factual and Social conditions.

A follow-up analysis of Condition differences at each 6-month increment revealed that the difference between the Factual and Social conditions emerged at 7.5 years of age. Before age 7.5, there was no significant difference between Conditions, *p*s_adj_ > .09. Starting at age 7.5, however, children in the Factual condition were significantly more likely to choose the *Mind* robot over the *Heart* robot compared to the Social condition, *p*s_adj_ < .004. This difference is explained by children starting to choose the *Mind* robot significantly above chance in the Factual condition (all estimated probabilities > 63.3%) at 7.5. years old, *p*s_adj_ < .006, while choices in the Social condition did not significantly differ from chance at all ages tested, *p*s_adj_ > .12.

#### Mind vs. Body.

**Figure 8.**
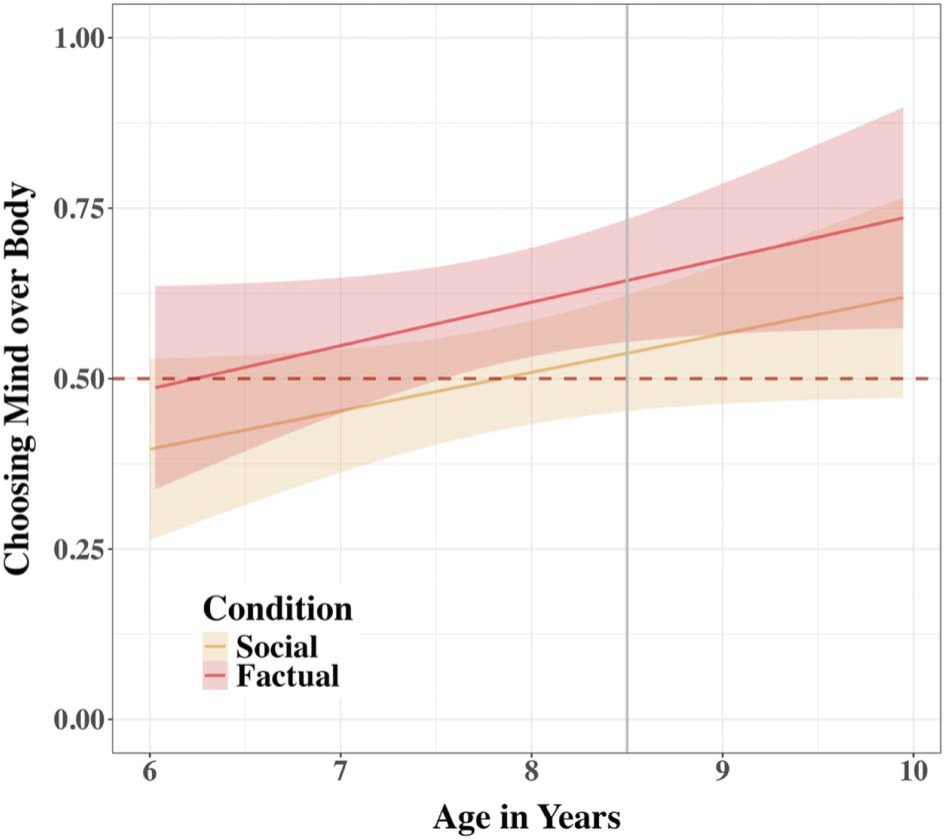
Tendency to Choose Mind over Body by Condition and Participant Age in Study 2. *Note*. A linear graph shows children’s increasing with age tendency to trust the *Mind* robot over the *Body* robot, averaged across trials across conditions in Study 2. Error around the regression line are 95% CIs. The horizontal line at 0.5 indicates a chance level of trust. The vertical line at 8.5 indicates the start of the significant difference between Conditions.

There was neither an interaction between Condition and age, *χ*^2^(1) = 0.04, *p*_adj_ = .843, nor an effect of Condition on the tendency to choose the *Mind* robot over the *Body* robot, *χ*^2^(1) = 3.06, *p*_adj_ = .181. There was, however, a significant effect of age, *χ*^2^(1) = 6.08, *p*_adj_ = .0495, indicating that for each additional year, the odds of choosing the *Mind* robot increased by a factor of OR = 1.28, 95% CI [1.05, 1.55] (Figure 8). A follow-up analysis at each 6-month increment revealed that participants selected the *Mind* robot over the *Body* robot significantly above chance starting at 8.5 years of age (all estimated probabilities > 59.3%), *p*s_adj_ < .018.

#### Heart vs. Body.

**Figure 9.**
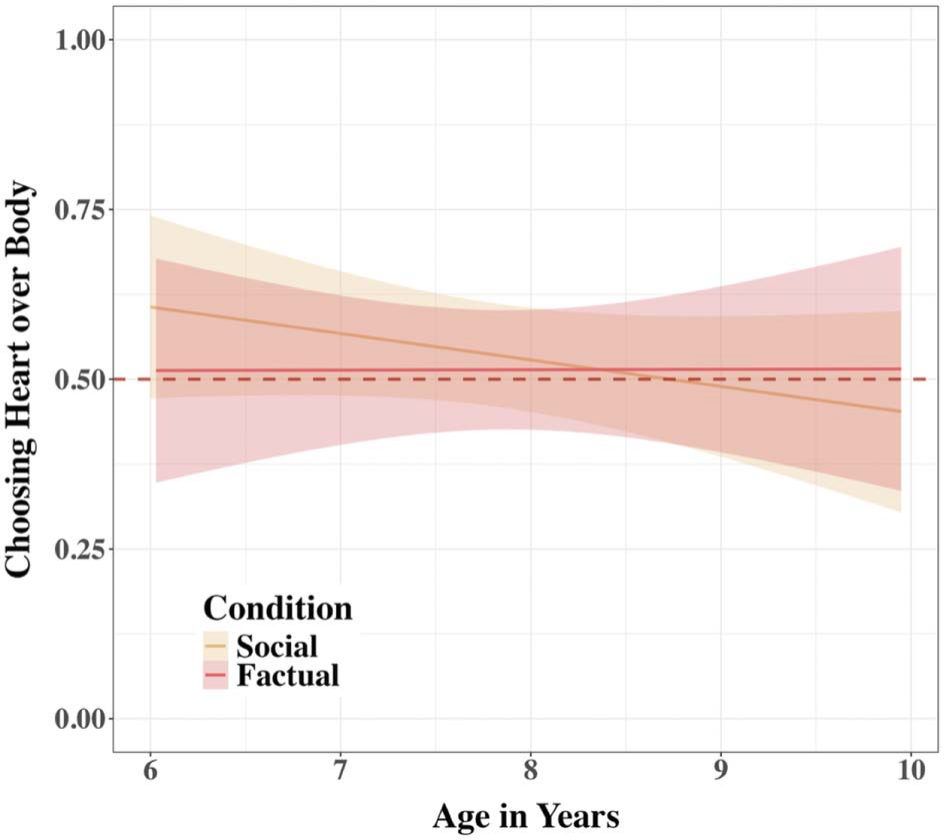
Tendency to Choose Heart over Body by Condition and Participant Age in Study 2. *Note*. A linear graph shows no effects of Condition or age on children’s tendency to trust the *Heart* robot over the *Body* robot, averaged across trials in Study 2. Errors around the regression lines are 95% CIs. The horizontal line at 0.5 indicates a chance level of trust.

There was no interaction between Condition and participant age, *χ*^2^(1) = 0.67, *p*_adj_ = .533, no main effect of participant age, *χ*^2^(1) = 0.72, *p*_adj_ = .533, and no main effect of Condition, *χ*^2^(1) = 0.12, *p*_adj_ = .826, on the tendency to choose the *Heart* robot over the *Body* robot (Figure 9). Across conditions and ages, children showed no preference to select either robot over another (estimated probability = 52.4%), *p* = .415.

### Discussion

Taken together, these results suggest that children’s trust in robots with different kinds of minds develops gradually and in context-dependent ways. Children endorsed the *Mind* robot over the *Heart* robot in a factual, but not a social, task. In the social task, children selected the *Mind* and *Heart* robots at equal rates. Selective factual trust in the *Mind* robot emerged at 7.5 years and became stronger with age, which is consistent with developmental trends reported in Study 1b as well as in past work on expertise compartmentalization (e.g., Danovitch & Keil, 2007, 2008) and mind perception (e.g., Weisman et al., 2017a, 2018). Moreover, children age 8.5 and older showed a growing tendency to trust the *Mind* robot over the *Body* robot across the board, further highlighting its overall epistemic advantage revealed by Studies 1a and 1b. In contrast, children chose the *Heart* and *Body* robots at equal rates across contexts and at all ages. All in all, the most robust effect from Study 2 is children’s selective trust in the *Mind* robot when it came to labeling objects.

## GENERAL DISCUSSION

As AI and robotics continue to explode and cater their efforts toward children, it becomes critical to understand whether a robot is like any other from a child’s point of view. Across three studies, we found evidence for children’s differential evaluations of (Studies 1A and 1B) and selective trust in (Study 2) robots with different types of mental capacities: cognitive-perceptual (the *Mind* robot), social-emotional (the *Heart* robot), and bodily/physical (the *Body* robot). Children’s patterns of responses revealed a strong and growing preference for the *Mind* robot in areas aligned with more traditional educational contexts (i.e., evaluations of intelligence, desire to learn from a robot, and learning novel words). When it came to social areas (i.e., evaluations of benevolence, desire to affiliate with, and social imitation), on the other hand, children’s responses were less clearcut, as they sometimes showed a preference for the *Mind* robot and sometimes for the *Heart* robot. Altogether, these findings shed new light on how different types of minds influence children’s evaluations of, and trust in, emerging AI technologies; they also open new directions for future research on developing folk philosophy of mind, naïve epistemology, and social cognition.

### Shedding New Light on Anthropomorphism: Minds Matter

Children’s tendency to anthropomorphize robots has been the main subject of developmental research on child–robot psychology (e.g., Goldman et al., 2025; Kahn et al., 2011; Kim et al., 2019; Okanda et al., 2021; Tung, 2016). Often, it is robots’ embodiment that has received the most attention and has been found to influence children’s tendencies to anthropomorphize these technological artifacts (e.g., Brink & Wellman, 2020; Caruana et al., 2023; Flanagan et al., 2023; Manzi et al., 2020). Although all robots had the same humanoid embodiment in our studies, children’s ratings of the robots’ similarity to themselves were sensitive to the robots’ mental capacities. This suggests that robots’ perceived minds, not embodiment itself, may be critical for children’s readiness to anthropomorphize them. Elaborating on past work that showed that presenting robots as lacking all psychological capacities decreased children’s perceptions of robots’ similarity to oneself (van Straten et al., 2023; van Straten, Peter, Kühne, et al., 2020), the present findings demonstrate that having at least one kind of human-like mind (*Mind*, *Heart*, or *Body*) is enough to increase children’s perceptions of robots’ similarity to humans. Strikingly, each type of mind increased this perception to the same degree.

This finding raises the question of whether there are different mechanisms behind comparable ratings of similarity for differently-minded robots. The physiological capacities associated with the *Body* may be seen as foundational for more advanced mental capacities (Weisman, Dweck, et al., 2021), especially among younger children (Weisman et al., 2018). A robot capable of embodied feelings may therefore seem similar by virtue of sharing such fundamental bodily essence with humans (Fiske & Haslam, 2005). When it comes to the *Mind* or *Heart* robots, their perceived similarity may stem from perceived human uniqueness or human nature (Haslam & Loughnan, 2014). Among traits viewed as uniquely human are the *Mind*’s rationality and logic as well as the *Heart*’s moral sensibility (e.g., knowing what is right and wrong) (Haslam, 2006). Human nature likewise includes *Mind*-like (e.g., agency, cognitive openness) and *Heart*-like capacities (e.g., emotional responsiveness, interpersonal warmth) (Haslam, 2006). Future studies could examine whether the perceptions of shared essence, human uniqueness, and/or human nature mediate children’s social judgments of robots to illuminate how children’s developing folk philosophy of mind and social cognition interact.

### Robots as Educational Partners: Cognition Matters

One of the most consistent patterns that emerged from the three studies reported here is children’s preference for the *Mind* robot in the context of educationally relevant judgments. Not only was the *Mind* robot rated as more intelligent than every other robot, but children also showed greater desire to learn from it compared to the other robots. Critically, the tendency to view the *Mind* robot as the smartest emerged reliably by age 7 and became stronger with age, shortly followed by the emergence of the explicit desire to learn from it around 7.5–8 years of age. By contrast, the *Heart* and *Body* robots did not differ from each other on either their perceived intelligence or epistemic appeal and were both rated lower than the *Mind* robot.

This developing epistemic advantage granted to the *Mind* robot was further evident in children’s selective trust in robots who told them “facts” (i.e., novel object labels). The preference for the testimony of the *Mind* robot over the *Heart* and *Body* robots emerged around 7.5–8 years of age and became stronger with age. These findings might be best understood within the context of children’s developing understanding of expertise. Whereas even young children have a sense of knowledge domains (e.g., Danovitch & Keil, 2004; Keil et al., 2008; Landrum et al., 2013), it is between ages 6–9 that children develop a more coherent understanding of distinct areas of expertise (e.g., Danovitch & Keil, 2004, 2007, 2008; Keil et al., 2008; Landrum & Mills, 2015). Indeed, during this period of development, children begin to group expert knowledge based on underlying principles (Keil et al., 2008) rather than superficial elements such as goals or topics (Danovitch & Keil, 2004). Our findings raise the possibility that an informant’s particular mental capacities may constitute one such underlying principle of expertise perception.

The revealed association between cognitive-perceptual mental capacities and epistemic expertise could be due, in part, to characteristics that children infer from them. Cognitive capacities (e.g., memory, problem-solving, language; Weisman et al., 2017b) might convey intelligence, competence, and knowledge, as they appeared to do in our studies. Children tend to trust intelligent and competent informants with science (Danovitch & Keil, 2007), pure facts (Danovitch & Keil, 2008), and object labels (Johnston et al., 2015). Although some work has shown that children’s trust is sensitive to informants’ benevolence even in factual domains (e.g., Danovitch & Keil, 2007; Landrum et al., 2013, 2015; Mascaro & Sperber, 2009; for a review, see Marble & Boseovski, 2020), our studies show that, despite being consistently rated as the nicest, the *Heart* robot did not hold such epistemic privilege, even when compared to the *Body* robot.

### Robots as Social Companions: Cognition and Emotion Matter

When it comes to robots’ potential as social companions, the picture seems to be more complex. Children did not simply grant social privilege to the *Heart* robot despite its seeming *a priori* social appeal. Rather, children were sensitive to both the robots’ capacities for cognition and emotion as evidenced by children’s desire to befriend, socially imitate, and view the robots as benevolent agents.

The equal preference for *Mind* and *Heart* robots was evident in children’s explicit desire to befriend them. Although past work has shown that children are generally friendly with robots (e.g., Fior et al., 2010; Kahn et al., 2013; Tung, 2016), it has largely assumed that children are influenced by robots’ external characteristics, such as appearance or behaviors (Hashimoto et al., 2013; Henkel et al., 2017), or it has simply measured their reactions to a particular robot (Fior et al., 2010; Kahn et al., 2012). Our findings qualify this past research by showing that a robot’s perceived mental capacities, beyond its humanoid appearance, are a crucial factor in children’s desire to form a potential friendship. Overall, children’s desire to befriend the robots declined with age, but a robot’s mind type exerted a strong influence, nonetheless. Across ages, all “mindful” robots elicited a greater affiliative desire than the robot with no mental capacities, but the *Mind* and *Heart* robots were further seen as more friendship-worthy than the *Body* robot.

A similar pattern emerged in the social trust task, which required participants to imitate one of the robot’s (pro)social behaviors. In stark contrast to children’s performance on the factual task, children of all ages in the social task showed no preference for either the *Mind* robot or the *Heart* robot. Moreover, children showed no selective tendency to socially imitate the *Heart* robot compared to the *Body* robot, suggesting that they may treat all kinds of feelings as equally relevant, or perhaps irrelevant, to their trust choices. These results, however, need to be interpreted with caution due to several limitations of Study 2, such as potential ambiguity regarding what each robot could not do and the lack of comprehension checks.^1^ Therefore, future work is needed to shed further light on these null findings. One direct step for future research would be examine what other factors, be they individual or contextual, might modulate children’s preference for technological companions capable of cognition or feeling in the social domain.

Nevertheless, the *Heart* robot was rated as nicer than all others, and this benevolence advantage only became stronger with age. This raises an intriguing possibility that robots with emotions might be influential in an area of great import—moral development (Constantinescu et al., 2022). Past research has demonstrated that capacities associated with *Heart* are frequently seen as morally important by adults (Schmittat & Burgmer, 2020) and that children prefer emotionally-capable moral advisors over intelligent ones (Danovitch & Keil, 2008). That said, moral expertise may also require capacities associated with *Mind* (Driver, 2006; Jones & Schroeter, 2012; Schmittat & Burgmer, 2020; Singer & Wells, 1984). In fact, children showed a preference to trust the *Mind* robot over the *Body* robot on the factual and social tasks alike, suggesting that beliefs about robots’ intelligence and epistemic potential may translate into children’s desire to learn from a robot on social tasks as well. Future work could combine our method of manipulating robots’ minds with a validated measure of moral trust (Li et al., 2019) to examine an intersection of mind and moral expertise perception (Li & Koenig, 2023), an issue that is relevant to long-standing debates about the nature of morality (Hume, 1739/1978; Kant, 1785/1997).

### Relationship Between Mind Perception and Trust in Technology

Overall, our work offers a new angle on children’s ability to learn from cutting-edge technologies. Previous research has shown that children prioritize different characteristics of human informants when deciding whom to trust or learn from (for reviews, see Harris et al., 2018; Marble & Boseovski, 2020; Mills, 2013). Among such characteristics are one’s past accuracy (e.g., Corriveau et al., 2013; Koenig et al., 2004), knowledgeability (Sabbagh & Baldwin, 2001), similarity (e.g., Elashi & Mills, 2014; Jaswal & Neely, 2006; Kinzler et al., 2011), benevolence and intelligence (e.g., Johnston et al., 2015; Landrum et al., 2013; Lane et al., 2013), and expertise (e.g., Kominsky et al., 2018; Lutz & Keil, 2002). One of these tendencies—accuracy—was shown to translate to children’s trust in robots, but support for whether children’s perceptions of robots’ minds play a part in this has been mixed (Brink & Wellman, 2020; Flanagan et al., 2024). Our studies provide evidence that robots’ different mental capacities influence both: children’s evaluations of characteristics known to affect trust and trust choices themselves.

What might explain these findings is children’s developing ability to differentiate the three dimensions of mind (Weisman et al., 2018). Children start to show an adult-like grasp of distinguishing between *Mind*, *Heart*, and *Body* starting around age 7 (Weisman et al., 2018). Therefore, children’s emerging views of the *Heart* robot as the most benevolent and the *Mind* robot as the most intelligent and trustworthy on matters of “fact” may reflect children’s growing dissociations within their folk conception of others’ minds. In contrast to earlier work showing that 7- to 9-year-olds grasp the difference between *Heart* and *Body* (Weisman et al., 2017a, 2018), they did not distinguish between them in our trust tasks or on measures of intelligence and epistemic appeal. Perhaps children view emotional and bodily feelings as largely similar (or similarly irrelevant) for purposes of choosing “teachers,” as also suggested by their explicit evaluations. Alternatively, 6- to 9-year-olds’ differential perception of emotional and bodily feelings may be still immature to be consistently applied to other problems, such as social learning. To explore this possibility, future work could use the present paradigms with older children and adults.

Moreover, past work has suggested that, when evaluating technological informants, children employ some of the similar strategies to those they use with humans (Brink & Wellman, 2020; Danovitch & Alzahabi, 2013; Flanagan et al., 2024; Geiskkovitch et al., 2019; Li & Yow, 2024; but see Stower et al., 2021). Among them is a domain-restricted allocation of trust: children show a preference for advice from technology when learning pure facts (Danovitch & Keil, 2008; Girouard-Hallam & Danovitch, 2022), about history or some domains of science (Wang et al., 2019) and machines (Oranç & Küntay, 2020), but not when they seek information that is personal (Girouard-Hallam & Danovitch, 2022), emotional and moral (Danovitch & Keil, 2008), or concerning human-centered science such as biology or psychology (Oranç & Küntay, 2020). Our studies suggest that such domain-constrained preferences could have emerged due to children’s preconceived notions of what type(s) of mind these technological devices possess. Indeed, at the age when children begin to appreciate different domains of mental capacities, they started to show high epistemic regard for the *Mind* robot, which is capable of cognition.

### Future Directions and Limitations

The work presented here was limited to one type of factual domain (i.e., learning novel object names) and one type of social domain (i.e., imitating sharing behaviors). Although the present findings suggest a gradually emerging but ultimately strong epistemic preference for the *Mind* robot on matters of fact, they raise multiple questions about how domains of mind might interact with other domains of knowledge. To extend this line of research, future work could apply our basic design to other domains of expertise, including biology and health in which physiological capacities may be privileged.

In an attempt to unambiguously isolate the three domains of mind perception (Weisman et al., 2017b), we chose the items that were the most representative of each domain and the least overlapping with other domains based on existing research on mind perception (Weisman et al., 2017a, 2018). In addition, we tried to keep the balance between the descriptions’ completeness and brevity to sustain children’s attention over trials. These choices resulted in the exclusion of perceptual items from the *Mind* domain due to their possible overlap with the *Body* domain (e.g., sensing temperatures, hearing sounds). As children have been previously found to prefer those who have perceptual access to information (Castelain et al., 2016; Pillow & Weed, 1997) and perhaps perceptual capacities (Oranç & Küntay, 2020), future work should examine how the presence of perceptual capacities influences children’s epistemic trust relative to other mental capacities.

Furthermore, the *Heart* robot’s description included “knowing what’s nice and what’s mean” since this capacity is central to the *Heart* domain (Weisman et al., 2017a, 2017b). This raises the concern about possible priming effects—that is, participants may have rated this robot as the nicest simply due to hearing the word “nice.” We therefore examined whether children’s ratings of niceness were affected by how soon this question was asked after the robot was introduced. These analyses revealed no effect of question order or interaction of question order with the type of mind a robot had, assuaging this concern to some degree (see Supplementary Materials).

To further generalize the trends observed here, future studies could validate the effects of mind perception by manipulating the medium of expression of mental capacities as well as the kinds of entities that express them. We focused on one explicit way to elicit mind perception via verbal descriptions of the contents of the robots’ minds. However, there are other ways to arrive at mind perception, for instance, by observing and/or interacting with a robot and subsequently inferring its capacities. To ensure that our findings generalize across different ways of arriving at dimensional mind perception, it is worth exploring whether other ways of eliciting comparable attributions produce similar consequences as the ones reported here.

To test whether these results stem from mind perception rather than from robot-specific perception, future work could examine how dimensional minds of other entities (e.g., human beings, various technologies, God, collectives) influence children’s evaluations. That said, not only focusing on the minds of AI and robots seems to be the most straightforward and plausible way of studying the consequences of diverse minds (Grigoreva et al., 2024; Wykowska, 2021), but it also paves the way for many timely questions as humans, including the youngest among us, grapple with the emergence of these technological companions.

Perhaps the biggest takeaway from this series of studies is that children’s social and epistemic evaluations of robots are sensitive to the types of human-like mind that robots have—humanoid embodiment on its own may not be enough. That said, our study did use a humanoid robot, Nao, which has been particularly popular with researchers (for a review, see Belpaeme, Kennedy, et al., 2018). Despite its child-friendly appearance and potential to transform children’s educational and social spaces, the availability of Nao is unlikely to match the availability of its disembodied counterparts (Auxier et al., 2020). Indeed, some of the latest emerging intelligent machines lack a body altogether. Among them are voice assistants, such as Siri or Alexa, and most recently large language models (LLMs) such as ChatGPT. Despite lacking a human-like body, or any type of body for that matter, these disembodied technologies have the potential to demonstrate a wide range of human-like mental capacities via linguistic and other symbolic communication.

The emergence of such technologies raises many timely and intriguing questions: Is embodiment necessary and/or sufficient for the perception of particular types of mental capacities? How do AI’s mind and body interact when it comes to children’s social and epistemic evaluations? Will new generations reimagine what it means to have certain mental capacities (e.g., feeling hungry; feeling love) when frequently confronted with disembodied, yet “mindful,” agents? Versions of such disembodied AI have already been shown to offer some of the social and educational benefits that have been previously examined in the context of robots (Hong et al., 2024; Liang et al., 2024; Liu et al., 2022). The methodology used in our studies, which revealed the importance of robots’ mental capacities, lends itself well to studying how different kinds of mental capacities expressed by disembodied AI may help or hinder children’s educational and social journeys.

### Conclusion

With the growing diversity of technological assistants in children’s lives, robots among them, understanding how children think about these different types of entities is essential. This work sheds new light on how robotic minds shape children’s social and epistemic judgments. Not only does this line of research illuminate what kinds of intelligent machines are most likely to appeal to children, but it also provides a new angle on long-standing questions about humans. By exploring how we view emerging technologies endowed with different aspects of humanness, we can also address what it is that we value about our own (human) minds.

## ACKNOWLEDGMENTS

The authors would like to thank the editor and two anonymous reviewers for their feedback on this work. We also thank the children and families that participated in our research, as well as the staff at Kelley Elementary School. We are also grateful to Stella Lourenco and Philippe Rochat for their feedback on this work, and to Cathy Cheng, Irene Cho, Elliana Nath, and Kylee Novick for help with data collection.

## AUTHOR CONTRIBUTIONS

Anastasiia D. Grigoreva Crean: Conceptualization; Data curation: Formal analysis; Investigation; Methodology; Project administration; Visualization; Writing – original draft, Writing – review & editing. Arber Tasimi: Conceptualization; Formal analysis; Funding acquisition; Methodology; Resources; Supervision; Writing – review & editing.

## DATA AVAILABILITY STATEMENT

All data are available at https://researchbox.org/4269.

## ETHICS APPROVAL STATEMENT

The research was approved by the Emory University Institutional Review Board (STUDY00002822: “Understanding social reasoning in adults and children”).

## Note

^1^ Our original submission of this article included what is now Study 1A and Study 2. Study 1B was conducted in response to the original set of reviews that we received.

## Supplementary Material


