# Supplementary material for: The Minds That Matter: How Robots’ Mental Capacities Shape Children’s Evaluations and Trust
Source: Open Mind (Camb). 2025 Oct 29;9:1550–75. doi: 10.1162/OPMI.a.37 (PMC12543304; doi:10.1162/OPMI.a.37)
Supplement: Supplementary file 1 [file opmi-09-1550-s001.pdf]

The Minds that Matter:

How Robots' Mental Capacities Shape Children's Evaluations and Trust

Supplementary Materials

1. Study 1a

1.1. Question Order

To determine whether there were any question order effects, we ran linear mixed-effects regression models with question order, Mind Type, and their interaction as fixed effects, and with a random intercept for participant to account for the within-subjects design. There was no main effect of order for any of the dependent variables: similarity, $\chi^2(3) = 1.32$, $p = .724$; willingness to be friends, $\chi^2(3) = 2.87$, $p = .412$; niceness, $\chi^2(3) = 2.96$, $p = .398$; or willingness to learn, $\chi^2(3) = 3.05$, $p = .384$. There was also no interaction between Mind type and question order for ratings of similarity, $\chi^2(9) = 5.45$, $p = .794$, or niceness, $\chi^2(9) = 15.04$, $p = .090$.

There was, however, an interaction between Mind Type and question order and for willingness to be friends, $\chi^2(9) = 22.20$, $p = .008$, and willingness to learn, $\chi^2(9) = 28.25$, $p < .001$. The analyses of estimated marginal means revealed that, in both cases, the differences lay in the *Control* condition. Children expressed less willingness to be friends with the *Control* robot when the question came first rather than second, $p_{adj} = .004$, or fourth, $p_{adj} = .019$; all other comparisons were not significant, $ps_{adj} > .120$. Likewise, children expressed less willingness to learn from the *Control* robot when the question came first rather than second, $p_{adj} = .014$, or third rather than fourth, $p_{adj} = .013$, and more willingness to learn from the *Control* robot when the question came first compared to third, $p_{adj} = .035$, and second compared to third, $p_{adj} < .001$; there were no other significant pairs, $ps_{adj} > .051$. Altogether, these order effects do not appear to be particularly meaningful given the lack of clear directionality in addition to the fact that they were present only for the *Control* robot, for which children's intuitions were likely the weakest, at least relative to the other three robots.

2. Study 1b

2.1. Question Order

To determine whether there were any question order effects, we ran linear mixed-effects regression models with question order, Mind Type, and their interaction as fixed effects, and with a random intercept for participant to account for the within-subjects design. There was no main effect of order for any of the dependent variables: similarity, $\chi^2(4) = 2.10$, $p = .718$; willingness to be friends, $\chi^2(4) = 2.60$, $p = .626$; niceness, $\chi^2(4) = 1.28$, $p = .865$; willingness to learn, $\chi^2(4) = 4.02$, $p = .404$; or smartness, $\chi^2(4) = 1.87$, $p = .760$. There were also no interactions between Mind type and question order for any of the dependent variables: similarity, $\chi^2(12) = 11.56$, $p = .480$; willingness to be friends, $\chi^2(12) = 6.51$, $p = .888$; niceness, $\chi^2(12) = 15.12$, $p = .235$; willingness to learn, $\chi^2(12) = 11.69$, $p = .471$; or smartness, $\chi^2(12) = 13.58$, $p = .328$.

2.2. Comprehension Check

Table S1

Comprehension Check Results by Condition and Age Group

**Mind Type**	**Age Group**<br>*Young: 6–7-year-olds*<br>*Old: 8–9-year-olds*	**No corrections on any trials**	**One correction on at least one trial**	**Two corrections on at least one trial**
Control	Young	89%	7%	4%
	Old	92%	8%	0%
Mind	Young	63%	37%	0%
	Old	90%	10%	0%
Heart	Young	53%	43%	4%
	Old	66%	34%	0%
Body	Young	57%	41%	2%
	Old	82%	18%	0%
**Total**	**Young**	**65%**	**32%**	**3%**
	**Old**	**82.5%**	**17.5%**	**0%**
